# Anatomical Models versus Nontactile Distanced Learning in Otolaryngology Teaching

**DOI:** 10.1055/s-0041-1733992

**Published:** 2021-09-14

**Authors:** Aashish Pandya, Dylan Mistry, David Owens

**Affiliations:** 1 Medical Education Department, School of Medicine, Cardiff University, Cardiff, United Kingdom; 2 Medical Education Department, University Hospital Wales, Cardiff, United Kingdom

**Keywords:** medical education, anatomy, tactile learning, distanced learning, models, otolaryngology

## Abstract

**Introduction** Medical schools in the United Kingdom are under increasing pressure to provide more streamlined, applicable teaching due to rising numbers of trainee doctors but are failing to meet their educational need for otolaryngology. The recent novel coronavirus disease 2019 (COVID-19) pandemic has placed additional pressures on medical schools to adapt the medium over which the curriculum is delivered. The use of tactile learning with three-dimensional models and distanced learning via videoconferencing may provide alternative teaching methods to meet otolaryngology undergraduate learning requirements. This pilot study aimed to assess the differences in undergraduate student attitudes toward tactile learning via nontactile distanced learning and review their acceptability among this cohort.

**Methods** Two groups of medical students observed a single educational event on the larynx and management of the airway. The learning opportunity was delivered in a lecture format with the lecturer demonstrating on an anatomical model of the larynx. Group one (tactile group) had an identical model to interact with during the lecture and were present within the lecture theater; group two (nontactile group) did not and observed the lecture via video link. Students were asked to rank their opinion to several statements about the session based on an 11-point Likert's scale and give qualitative feedback.

**Results** All ranked feedback was mainly positive. Tactile learning was statistically equivalent to nontactile learning based on the ranked feedback from the students, except for “improvement in anatomical knowledge,” for which the students believed tactile learning was superior ( *p* = 0.017). A variety of qualitative feedback was received by both groups.

**Conclusion** This pilot study provides evidence for the acceptability among students of the use of nontactile distanced learning to deliver the otolaryngology undergraduate curriculum compared with tactile learning. This can provide the basis for larger studies to assess the educational impact of these different teaching methods.

The General Medical Council's (GMC) 2011 to 2013 education strategy 1 requires medical education institutions to train and produce doctors with appropriate knowledge and skills, in an appropriate environment and by a suitable trainer. The Government's plan to increase the number of medical students in the United Kingdom (UK) along with GMC guidance 1 has put pressures and ultimately changed the medical curriculum. 2 Modern day curricula are aimed to reduce factual overload, decrease expenses, and make teaching more clinically orientated. 3 Medical schools are struggling to meet these requirements in general, especially when it comes to otolaryngology undergraduate training. 4 5 6 Time and commitment restraints often make it difficult to provide a formal otolaryngology rotation and in some cases no rotation is offered at all. 6 In addition, the novel coronavirus disease 2019 (COVID-19) pandemic has led to cancellation of formal clinical placements, requiring medical schools to deliver sessions via online platforms. 7

The amount of otolaryngology exposure in the undergraduate curriculum has been a concern for over 30 years. 4 8 This is especially worrying given that otolaryngology is the sixth largest surgical specialty, with one in sixth of general practice (GP) consultations being otolaryngology related and forming 50% of pediatric presentations. 9 10 11 A systematic review by Ferguson et al 5 found final year medical students and first year foundation doctors had a lack of confidence and ability in being able to manage otolaryngology patients. 4 12 13 This has been attributed to a lack of undergraduate exposure to otolaryngology, with questions raised over the suitability and use of teaching materials. 4

A key component in becoming more confident in managing otolaryngology cases is a firm grasp of the anatomy. Anatomy has become the greatest causality of the modern curriculum with cadaveric dissection being removed, reduced, or limited to prosection-based teaching. 14 Given the reduction in anatomy input in the modern medical curriculum, it is important to explore other methods of teaching.

There is clear evidence that three-dimensional (3D) models are superior for student learning and experience for anatomical demonstrations, particularly when demonstrating complex spatial relationships. 15 Their implementation in the medical curriculum is feasible 16 but is currently scarce, largely due to their financial burden. When delivering learning outcomes based on a curriculum, no single teaching modality can meet all aspects of the curriculum and a combination is essential to address them all. 16 17 Students at the same medical school can be placed over a large geographical area within different hubs, making it difficult to standardize the teaching they receive, particularly when using the traditional face-to-face methods. Advances in technology and the World Wide Web has helped breakdown the barrier imposed by geographical distance between teachers and students. In contrast, videoconferenced distance learning allows students to interact with sessions in real time regardless of geographical location. 18 19 A combination of 3D physical models with technology could provide a more effective learning experience for otolaryngology anatomy, physiology, and management.

The aim of this pilot study was to assess and compare student opinions on tactile teaching compared with nontactile videoconferencing, in the context of otolaryngology undergraduate training using learning outcomes from the Students and Foundation Doctors in Otolaryngology (SPO-UK) curriculum for the larynx ( Supplementary Appendix 1 ; available in the online version). Its secondary aims was to assess the feasibility of organizing and delivering these teaching sessions for larger studies in future.

## Methods

### Study and Learning Session Design

A cohort study was developed with two groups observing a single educational event on the larynx and management of the airway provided by an otolaryngology registrar. The learning opportunity was delivered in a lecture format with the lecturer using an anatomical model of the larynx ( Supplementary Appendix 2 ; available in the online version) as a fulcrum to the lecture. Two learning groups were created. Group one (tactile group) had an identical model to the lecturer to interact with during the lecture. Group two (nontactile group) did not have this model to interact with but were able to observe the model used by the lecturer. The tactile group were present in the same study room as the lecturer. The participants in the nontactile group had the slides streamed to them via videoconferencing. This meant one group was taught through tactile learning (the tactile group) and the other group was taught through nontactile videoconferencing (nontactile group).

The learning outcomes for the session ( Supplementary Appendix 1 ; available in the online version) were based on the SPO-UK undergraduate curriculum for the larynx. This included anatomy, function, and the clinical presentation and management of pathologies such as laryngitis, epiglottitis, vocal cord polyps and nodules, laryngeal edema. Procedures, such as tracheostomy and laryngectomy, were also covered in the presentation.

### Participant Recruitment

The participants were third year medical students who were undertaking a surgical attachment at a medical school in the UK but had not yet had any scheduled teaching on the larynx as part of their formal teaching associated with their attachment. The learning outcomes for their attachment shared some overlap with the learning outcomes used for this study. The study size aimed to be representative of the number of students typically observed in a teaching session, and was consistent with the numbers typically observed for a pilot study.

A teaching slot was timetabled at the same time at the undergraduate centers of the two placement hospitals. Students had previously been allocated to each center at random by the medical school.

### Data Collection

Participants were given a questionnaire after the teaching session ( Supplementary Appendix 3 ; available in the online version) asking them to rank their opinion from 0 to 10 (as per the 11-point Likert's scale), about nine statements aimed at assessing how valuable the sessions were for their undergraduate education. A score of 0 meant they strongly agreed with the statement, whereas a score of 10 was equivalent to strong disagreement. It also asked them to provide positive and negative qualitative feedback about the session.

The nine statements which were rated by the participants are as follows:

1. Session was useful.
2. Session met education needs.
3. Session met learning outcomes indicated at the start.
4. Overall I was satisfied with the session.
5. This session enhanced my ENT (ear–nose–throat) learning experience.
6. The session has improved my anatomical knowledge.
7. The session encouraged me to learn more about head and neck anatomy.
8. The session encouraged me to learn more about ENT.
9. I would be willing to attend future sessions delivered in a similar manner.

### Statistically Analysis

Analysis of each statement's ranked data was performed using the Mann–Whitney *U* -test using IBM SPSS Version 25.0.0.0. Participant's written feedback was split into positives and negatives and was processed into a word map using Microsoft Word Version 16.35.

### Outcome Measures

The primary outcome was to assess whether tactile teaching is as effective as nontactile distanced learning for student satisfaction for learning as indicated on an 11-point Likert's scale during an otolaryngology undergraduate training session.

Secondary outcomes included the points mentioned below:

- To assess the positive and negatives of tactile compared with nontactile teaching from students receiving each type of teaching.
- To assess the ease of delivering tactile learning opportunity compared with a nontactile distanced learning session.
- To assess whether nontactile videoconferencing is a feasible teaching method compared with tactile teaching for otolaryngology anatomy, function, and management.

## Results

### Demographics

In a total of 22 participants attended the teaching session. Sixteen students signed up from the undergraduate center allocated to the nontactile teaching group. Six students signed up from the center allocated to tactile face to face group. Their demographics are demonstrated in Table 1 .

**Table 1:** Participant demographics

Descriptor	Numbers of participants
Participant numbers	22
Tactile face to face group	6
Non tactile videoconference group	16

### Participant Ranked Responses

Ranked data for each statement from the tactile group are shown in Table 2 ; and for the nontactile group in Table 3 . No participant disagreed with any statement from the tactile group. In the nontactile group, the majority agreed with most of the statements; however, every statement had at least one person disagreed.

**Table 2:** Ranked feedback from the tactile group

Statements	0	1	2	3	4	5	6	7	8	9	10
Session was useful	3		2			1					
Session met education needs	2		2	1	1						
Session met learning outcomes indicated at the start	4		1			1					
Overall I was satisfied with the session	3	1		2							
This session enhanced my ENT learning experience	3	1	2								
The session has improved my anatomical knowledge	5		1								
The session encouraged me to learn more about head and neck anatomy	2		3		1						
The session encouraged me to learn more about ENT	1	1	1	1	1	1					
I would be willing to attend future sessions delivered in a similar manner	2	1	1			2					

Abbreviation: ENT, ear–nose–throat.

Note: Values are the number of participants who ranked the statement for that specific score. 0 means strongly agree and 10 means strongly disagree.

**Table 3:** Ranked feedback from the nontactile group

Statements	0	1	2	3	4	5	6	7	8	9	10
Session was useful	5		5	3		1		2			
Session met education needs	4	2	2	4	1			3			
Session met learning outcomes indicated at the start	7	1	1	4				3			
Overall I was satisfied with the session	6	3		3	1			3			
This session enhanced my ENT learning experience	3	3	1	4		3	2				
The session has improved my anatomical knowledge	4	3		4	3	1		1			
The session encouraged me to learn more about head and neck anatomy	4	3	2	1	1	3		2			
The session encouraged me to learn more about ENT	4	3		4	1	2		2			
I would be willing to attend future sessions delivered in a similar manner	5	1	2		1	1	1	4		1	

Abbreviation: ENT, ear–nose–throat.

Note: Values are the number of participants who ranked the statement for that specific score. 0 means strongly agree and 10 means strongly disagree.

Table 4 shows a comparison of the median scores for each statement for the two groups. The tactile group's median scores were lower in all statements expect “The session encouraged me to learn more about head and neck anatomy.” “The session has improved my anatomical knowledge” was the only statement showing statistically significant between the two groups, with the tactile group agreeing more with the statement ( *p* = 0.017).

**Table 4:** Analysis of the median scores of each statement as ranked by participants for each group

Statement	Tactile group median (IQR) scores	Tactile group median (IQR) scores	*p* -Value
Session was useful	1.0 (0.00–2.75)	2.0 (0.00–3.00)	0.356
Session met education needs	2.0 (0.00–3.25)	2.5 (0.25–3.75)	0.573
Session met learning outcomes indicated at the start	0.0 (0.00–2.75)	1.5 (0.00–3.00)	0.322
Overall I was satisfied with the session	0.5 (0.00–3.00)	1.0 (0.00–3.75)	0.418
This session enhanced my ENT learning experience	0.5 (0.00–2.00)	3.0 (1.00–5.00)	0.051
The session has improved my anatomical knowledge	0.0 (0.00–0.50)	3.0 (0.25–4.00)	0.017
The session encouraged me to learn more about head and neck anatomy	2.0 (0.00–2.50)	2.0 (0.25–5.00)	0.475
The session encouraged me to learn more about ENT	2.5 (0.75–4.25)	3.0 (0.25–4.75)	1.000
I would be willing to attend future sessions in similar format to this	1.5 (0.00–5.00)	3.0 (0.00–7.00)	0.407

Abbreviations: ENT, ear–nose–throat; IQR, interquartile range.

### Participant Written Feedback

**Fig. 1:**
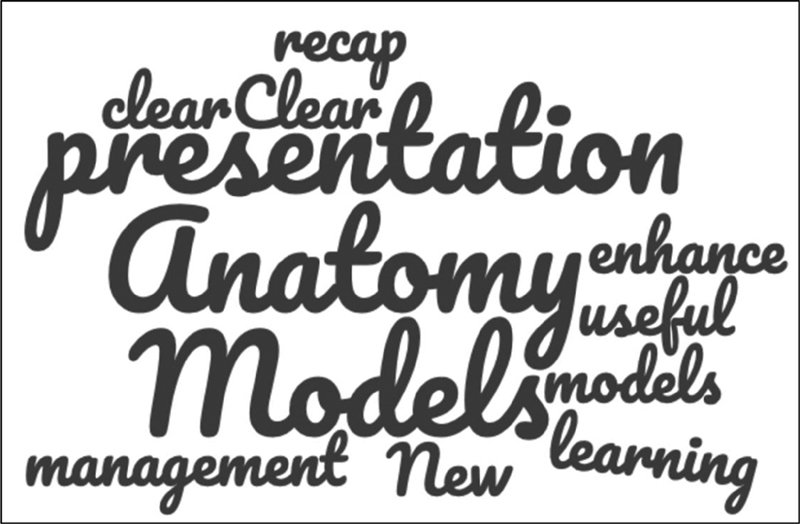
Positive comments received by tactile group.

**Fig. 2:**
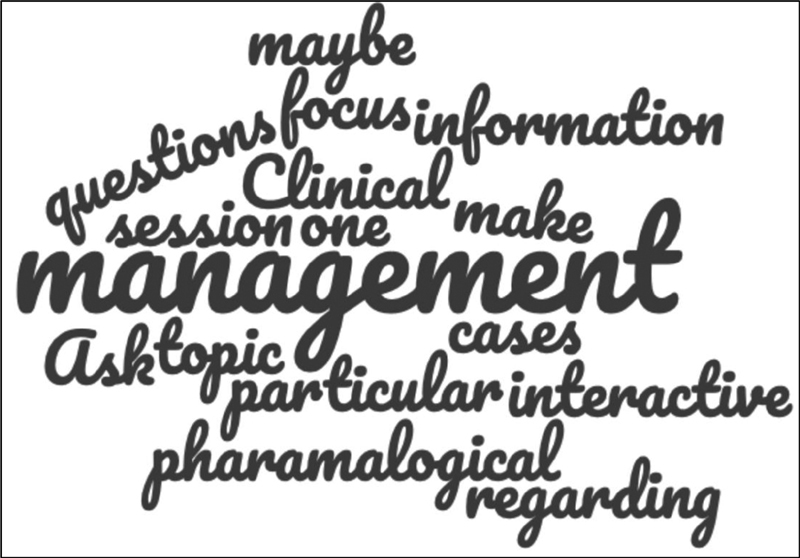
Negatives comments received by the tactile group.

**Fig. 3:**
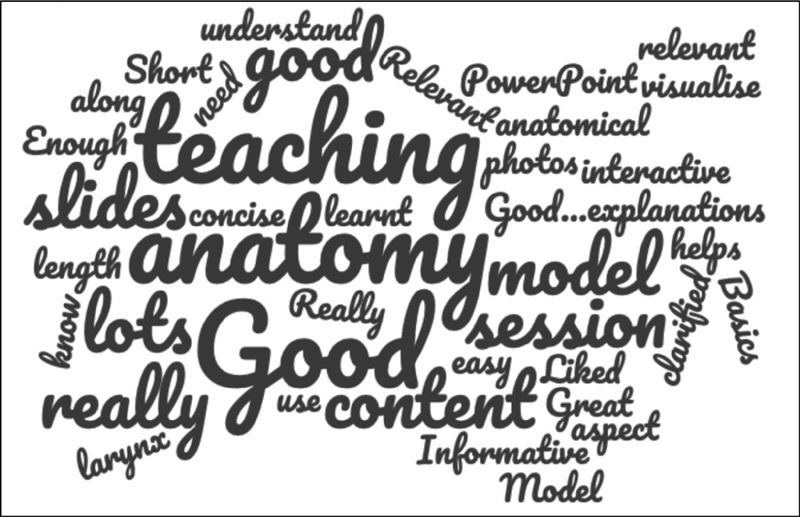
Positive comments received by the nontactile group.

**Fig. 4:**
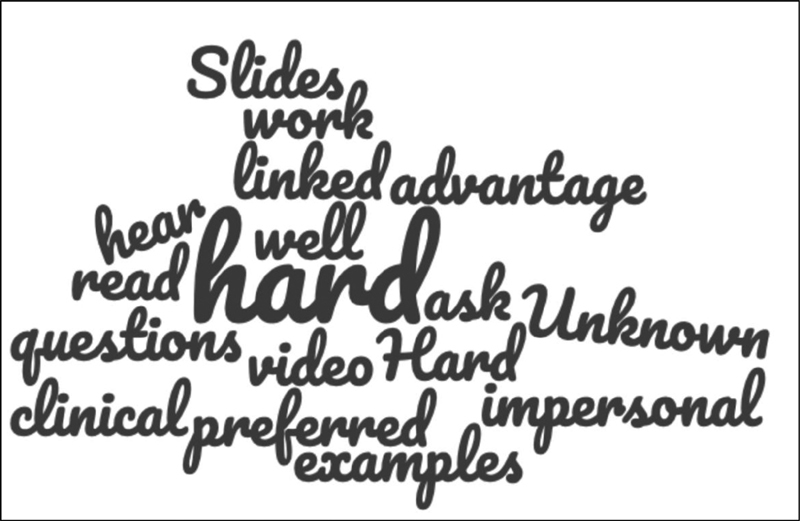
Negatives comments received by the nontactile group.

Written qualitative feedback is demonstrated by the word maps below for each group ( Figs. 1 , 2 , 3 , 4 ). The comments were split into positive and negative comments for the two groups.

The specific individual comments from the qualitative feedback can be found in Supplementary Appendix 4 (available in the online version).

## Discussion

This study endeavored to compare student satisfaction between tactile with nontactile videoconference learning in undergraduate otolaryngology training. Analysis between the tactile and nontactile groups shows equivalent results. Both groups had positive responses to the session, especially when considering usefulness, meeting educational needs, and attending future sessions in this format. This was also reflected in the positive qualitative feedback given by the students.

The only statistical difference between the two groups was a greater student satisfaction in the improvement of their anatomical knowledge seen in the tactile group. However, from the word clouds generated from positive student feedback ( Figs. 1 and 3 ), the most dominating word was anatomy which may suggest a beneficial anatomical learning experience to the nontactile group. The collation of this data shows that nontactile distanced learning is just as acceptable as tactile learning among students for delivering the otolaryngology curriculum.

While there was a significant difference in group sizes, this may be more akin to the practicalities of each session. In-person teaching sessions are limited by room sizes, availability of teachers, and students being able to physically attend sessions. Conversely, videoconferencing teaching sessions have the potential to be sent to a larger number of people, regardless of geographical location.

During a time when the medical curriculum is being subject to change, the use of videoconferencing and, in particular, nontactile learning could provide an alternative method of meeting educational requirements, given the constraints now faced by medical schools. 6 While there was no clear difference in the use of 3D models enhancing learning, the students in this study with access to the model believed it heightened their anatomical educational experience. This may be due to anatomical model's abilities to “offload cognition or free cognitive resources during learning.” 20

The findings from this study are in keeping with previous studies that assess tactile anatomy learning; however, this is the first to assess student opinion or acceptability and not objective assessments such as tests scores. Preece et al 15 demonstrated statistically higher test scores in students who used physical 3D models compared with computer 3D models and textbook images. The advantage of physical 3D models is the tactile manipulation which aids retention and understanding of spatial information and relationships. 21 22 This is also applicable to dissection which incorporates multiple senses when learning anatomy. Computer-based 3D models are unable to offer these advantages but still have their education benefits. 23 Alnabelsi et al 18 also found no difference between face to face and synchronous learning students' groups when comparing pre- and posttest scores in medical undergraduate otolaryngology teaching; however, this particular study did not include the use of models.

The use of physical 3D models in teaching anatomy does have its limitations. Models may be subject to damage, theft, misplacement, and reduction in quality over the years with constant use. 15 Physical anatomical models also have a large commercial burden, with multiple models often required to depict the organs and systems of the human body. Their price is compounded with maintenance and storage requirements, with higher quality models requiring greater care and security.

Videoconferencing also has its own constraints and relies on experience to run efficiently and first-time users may struggle. This could influence the satisfaction and learning experience of videoconference learning. It also requires good internet connection of the host and participants, without which it could lead to a poor learning experience.

## Limitations

This study does suffer from limitations. Its small sample size affects its impact and comparability with the broader medical community and recruitment relied on a single medical school's placement allocation process. Anatomical knowledge was not formally assessed as this would require quantitative analysis and data collection. This would need tests repeatedly performed over a prolonged period of time to evaluate information retention which was not part of this study design. This small “Pilot”-type study has merits in planning further research and identifying areas of potential future study. Moving forward, a bigger randomized sample over multiple sites with multiple medical school involvement would improve this study. Four learning modalities were investigated across just two groups. Ideally four following groups should have been used: (1) tactile face to face, (2) nontactile face to face, (3) tactile videoconferencing, and (4) nontactile videoconferencing. However, having a distanced learning group with access to a model may not be realistic. Objective educational measures such, as pre- and postsession test scores, could be used to assess quantitative educational impact of these teaching modalities.

## Conclusion

This pilot study demonstrates that tactile and nontactile learnings are both equally acceptable teaching styles based on student opinion for the delivery of the otolaryngology undergraduate training. The positive experiences were represented in quantitative and qualitative feedback. However, tactile learning was superior in improving student opinion on anatomical knowledge. These findings may provide an effective alternative method of teaching for anatomy and teaching sessions in general given the challenges faced by the modern medical school curriculum. This pilot study has shown that it is possible to organize and deliver these teaching sessions and provides a blueprint for further studies which can aim to further access tactile versus nontactile teaching which could be combined with distance learning. Future larger studies could integrate the use of quantitative analysis of repeated tests performed over a prolonged period of time to evaluate information retention. In addition, a longitudinal study could also be formulated to follow students into professional practice to determine if there are differences in clinical outcomes/practices based on learning modality.

Larger studies are required before these kind of teaching methods that can have a bigger impact on the wider medical community, especially given that there are significant positives and negatives to each teaching method. This small “Pilot”-type study has merits in planning future research and identifying solutions to shortcomings in the current medical curriculum.
