# Supplementary material for: Anatomical Models versus Nontactile Distanced Learning in Otolaryngology Teaching
Source: Surg J (N Y). 2021 Sep 14;7(3):e259–64. doi: 10.1055/s-0041-1733992 (PMC8440056; doi:10.1055/s-0041-1733992)
Supplement: Supplementary file 1 — Supplementary Material [file 10-1055-s-0041-1733992-s2100027oa.pdf]

Supplementary Material

Supplementary Appendix 1

The Students and Foundation Doctors in Otolaryngology (SPO-UK) curriculum learning outcomes

B. HEAD AND NECK

1. Be aware of the structure and function of the following (learn 1)(learn 2):

   a. Neck*
   b. Thyroid gland*
   c. Oral cavity*
   d. Pharynx and larynx
   e. Salivary glands

Have an awareness of how these structures change when affected by disease and understand how this leads to dysfunction and patient morbidity and mortality.

2. Understand the basic physiological principles of swallowing (learn)

3. Understand the incidence/prevalence, clinical presentations, the management and prognosis of the following malignant head and neck conditions:

   a. Carcinoma of the larynx*(learn 1)(learn 2)
   b. Carcinoma of the pharynx including nasopharyngeal carcinoma*
   c. Carcinoma of the oral cavity*
   d. Carcinoma of the oesophagus*
   e. Cutaneous malignancy of the head and neck eg. SCC, BCC, melanoma

Be aware that lymphoma can affect the head and neck region

A basic understanding of the modalities of treatment available for the treatment of head and neck cancer is required.

4. Be aware of the natural history of head and neck cancer and the TNM staging system

5. Be aware of head and neck red flag symptoms*.

6. Be aware of the risk factors for head and neck cancer and what means are available for preventing head and neck cancer*.

7. Be aware of current fast-track referral guidance. Further information can be found at:

www.nice.org.uk/nicemedia/pdf/cg027niceguideline.pdf
www.scotland.gov.uk/Resource/Doc/46922/0014162.pdf

8. Understand the incidence/prevalence, clinical presentations, the management and prognosis of the following benign head and neck conditions in adults:

   a. Acute tonsillitis*
   b. Peritonsillar abscess and parapharyngeal abscess (learn 1)(learn 2)*
   c. Epiglottitis*
   d. Laryngitis*
   e. Pharyngitis*
   f. Infectious mononucleosis*
   g. Obstructive sleep apnoea*
   h. Acute laryngeal oedema*
   i. Branchial cyst
   j. Lesions of the oral cavity including leukoplakia
   k. Vocal cord paralysis
   l. Vocal cord nodules and polyps
   m. Pharyngeal pouch (learn)

Have a broad understanding of how to manage acute airway obstruction (learn 1)(learn 2)

9. Be aware of the importance of laryngopharyngeal reflux* in the aetiology of diseases of the pharynx and larynx

10. Understand the incidence/prevalence, clinical presentations, the management and prognosis of the following head and neck conditions that present in children:

   a. Acute tonsillitis*
   b. Epiglottitis*
   c. Adenoid hypertrophy*
   d. Allergic chronic rhinosinusitis*
   e. Mumps*
   f. Croup
   g. Pertussis

11. Understand the incidence/prevalence, clinical presentations, the management and prognosis of the following thyroid conditions in adults:

   a. Graves disease*
   b. Multinodular goitre*
   c. Hypo and hyperthyroidism*
   d. Thyroid malignancy*
   e. Thyroiditis*
   f. Thyroglossal cyst

12. Understand the incidence/prevalence, clinical presentations, the management and prognosis of the following salivary gland conditions in adults:

   a. Benign and malignant salivary gland tumours*
   b. Sialadenitis
   c. Salivary gland stones and strictures
   d. Sjogren's syndrome
   e. Xerostomia

13. Have a basic knowledge of the following head and neck procedures:

   a. Tonsillectomy*
   b. Adenoidectomy*
   c. Tracheostomy

 © 2021. The Author(s).

Supplementary Appendix 2 Photos of the model used by the teacher during the session

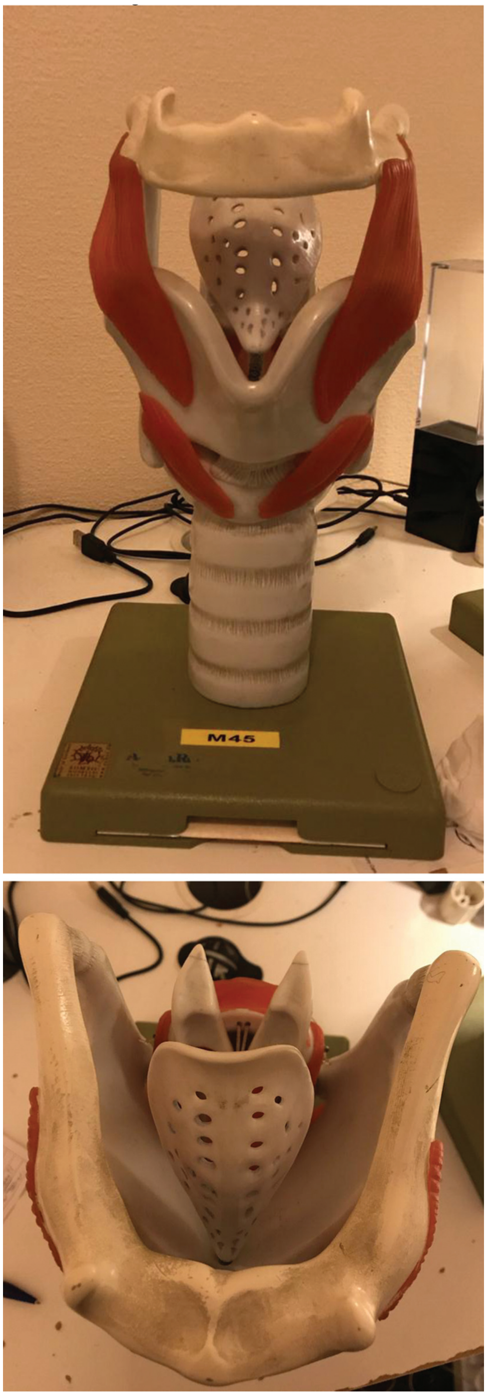

 © 2021. The Author(s).

Supplementary Appendix 3 A copy of the questionnaire given to students after the session

I. ENT teaching session on the larynx

TITLE OF TEACHING:

TEACHER: **DATE:**

Have you completed (or started) your ENT teaching sessions as part of the block? Yes ☐ No ☐

Please indicate on the scale from 1 to 10, the value which represents your opinion to the following statements:

1. I found the session useful

0 1 2 3 4 5 6 7 8 9 10

Strongly Agree | Neutral | Strongly Disagree

2. This session met my education needs

0 1 2 3 4 5 6 7 8 9 10

Strongly Agree | Neutral | Strongly Disagree

3. This session met the learning outcomes outlined at the start

0 1 2 3 4 5 6 7 8 9 10

Strongly Agree | Neutral | Strongly Disagree

4. Overall I was satisfied with the session

0 1 2 3 4 5 6 7 8 9 10

Strongly Agree | Neutral | Strongly Disagree

5. This session enhanced my ENT learning experience

0 1 2 3 4 5 6 7 8 9 10

Strongly Agree | Neutral | Strongly Disagree

 © 2021. The Author(s).

6. This session has improved my anatomical knowledge

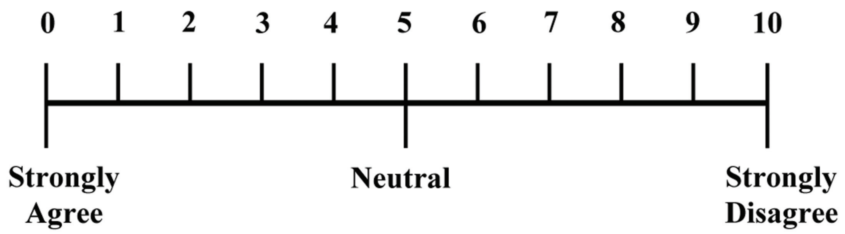

7. This session has encouraged me to learn more about head and neck anatomy

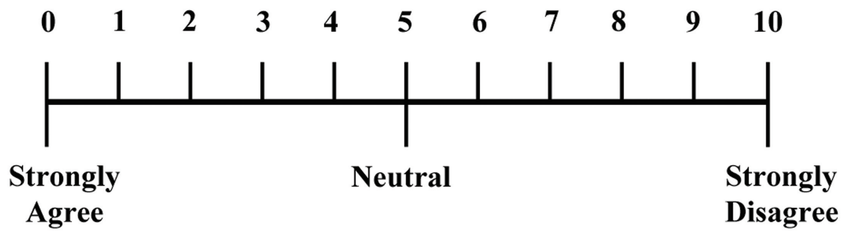

8. The session has encouraged me to learn more about ENT

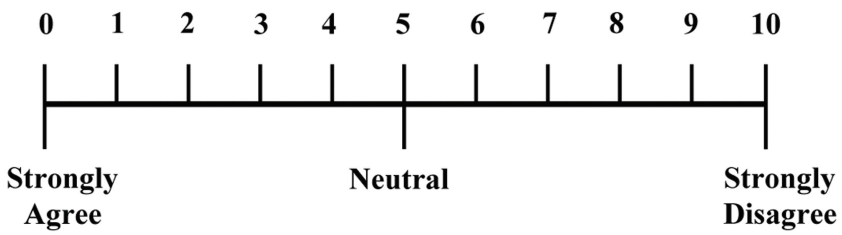

9. I would be willing to attend future sessions that were video linked

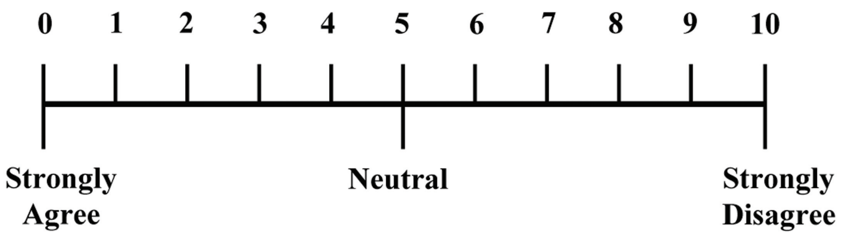

 © 2021. The Author(s).

Please answer the following questions on the session:

What did you like?

What didn't you like?

How could the session be improved?

 © 2021. The Author(s).

Supplementary Appendix 4 Qualitative feedback from the tactile and nontactile groups

Positive	Negative	Comments
Qualitative feedback from the nontactile group		
Anatomy models	Too short not testing	Ask students questions and make session interactive
Models to enhance learning		Slightly more information regarding management
Anatomy, pathologies, and management clearly explained		Nice overview, maybe focus on one particular topic
Good presentation, very useful		Clinical cases would be useful and go over management—pharmacological, as well as intubation
Models to explain anatomy		
Haven't had teaching on topic before		
Anatomy and clinical recap		
Qualitative feedback from the nontactile group		
Basics on anatomy	Slides were a bit too hard to read	Having someone demonstrate the model
Good teaching on anatomy of larynx Good length of presentation	Watching myself on video was a little strange	
Great teaching really relevant	Don't know if there was any advantage in having the session video linked	
The model really clarified the anatomical teaching	Slightly impersonal as we could not see his face	
PowerPoint was good. Enough content		
Model along with slides helps to visualize anatomy	Can be hard to hear	
The slides and interactive aspect of teaching		
Short and easy to understand		Done at a point where everyone has had or is having teaching
Good ... explanations.	More clinical examples	
Informative session	Video teaching did not work that well	More ENT teaching that is face to face
	Would have preferred not being video linked	
Really good session—learnt lots. Liked the anatomy model and lots of use of photos		Could be longer
Good content—concise and what you need to know	Bit weird being on video link as it feels funny to ask questions but would rather this than not	Case examples
Relevant		Presentation was quite hard to see as quite small on video link

 © 2021. The Author(s).
